# Functional Carbon Materials Derived through Hypergolic Reactions at Ambient Conditions

**DOI:** 10.3390/nano10030566

**Published:** 2020-03-20

**Authors:** Nikolaos Chalmpes, Georgios Asimakopoulos, Konstantinos Spyrou, Konstantinos C. Vasilopoulos, Athanasios B. Bourlinos, Dimitrios Moschovas, Apostolos Avgeropoulos, Michael A. Karakassides, Dimitrios Gournis

**Affiliations:** 1 Department of Materials Science & Engineering, University of Ioannina, 45110 Ioannina, Greece; chalmpesnikos@gmail.com (N.C.); asimakopoulos.geo@gmail.com (G.A.); konstantinos.spyrou1@gmail.com (K.S.); kovasil@auth.gr (K.C.V.); dmoschov@cc.uoi.gr (D.M.); aavger@uoi.gr (A.A.); mkarakas@uoi.gr (M.A.K.); 2 Physics Department, University of Ioannina, 45110 Ioannina, Greece

**Keywords:** hypergolic reactions, fuming nitric acid, carbon nanosheets, graphitic carbon nitride, photoluminescent carbon dots

## Abstract

Carbon formation from organic precursors is an energy-consuming process that often requires the heating of a precursor in an oven at elevated temperature. In this paper, we present a conceptually different synthesis pathway for functional carbon materials based on hypergolic mixtures, i.e., mixtures that spontaneously ignite at ambient conditions once its ingredients contact each other. The reactions involved in such mixtures are highly exothermic, giving-off sizeable amounts of energy; hence, no any external heat source is required for carbonization, thus making the whole process more energy-liberating than energy-consuming. The hypergolic mixtures described here contain a combustible organic solid, such as nitrile rubber or a hydrazide derivative, and fuming nitric acid (100% HNO_3_) as a strong oxidizer. In the case of the nitrile rubber, carbon nanosheets are obtained, whereas in the case of the hydrazide derivative, photoluminescent carbon dots are formed. We also demonstrate that the energy released from these hypergolic reactions can serve as a heat source for the thermal conversion of certain triazine-based precursors into graphitic carbon nitride. Finally, certain aspects of the derived functional carbons in waste removal are also discussed.

## 1. Introduction

**Scheme 1:**
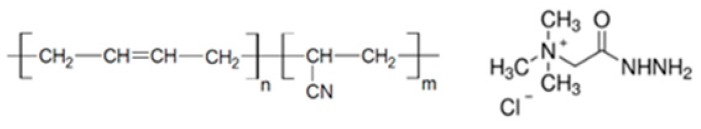
Molecular structures of the disposable nitrile glove (left) and Girard’s reagent T (right).

Hypergolic mixtures consist of two different substances that spontaneously ignite upon coming into contact at ambient conditions. One substance plays the role of a strong oxidizer (fuming HNO_3_, N_2_O_4_, H_2_O_2_, Cl_2_), whereas the other one the role of the combustible fuel (aniline, nitrile rubber, hydrazine derivatives, ionic liquids, acetylene) [1,2,3,4,5,6]. Such reactions are often used in chemistry demonstrations to show the impressive power of chemical energy, as well as ways to exploit it in real life applications (e.g., rocket propellants and fuels). The flames, smokes, sound, and motion produced by these reactions create a stunning result that captivates the senses of an observer. Interestingly, many of these reactions produce carbon, if organic fuels are used; surprisingly, in the literature, only little emphasis is given on this important aspect of hypergolic reactions [6]. Taking into consideration the central role of carbon in materials science and synthesis, hypergolic reactions can certainly provide an energy-saving and thermodynamically favoured alternative towards the direct synthesis of carbon materials at ambient conditions. In this respect, no external source of heat is required to carbonize the organic precursor, such as energy-consuming ovens operating at elevated temperature. As a matter of fact, hypergolic reactions release sizeable amounts of energy that could be further exploited in the production of useful work (mechanical, electrical, chemical, etc.) [7]. In addition, such reactions are kinetically favoured, thus allowing rapid product formation at room temperature and atmospheric pressure (e.g., within few seconds). The main purpose of this work is to highlight the importance of hypergolic reactions in the area of carbon materials synthesis. To this aim, we provide examples of hypergolic reactions that directly lead to the formation of different types of functional carbon materials at ambient conditions. Functional carbon materials include carbon nanosheets [8], graphitic carbon nitride [9], and photoluminescent carbon dots [10]. In one case, we show that the reaction of disposable nitrile gloves (acrylonitrile butadiene rubber) with fuming nitric acid (100% HNO_3_) results in carbon nanosheets, thus offering a way to transform this waste plastic into a valuable product. Moreover, the heat produced from the ignition of the glove can be further utilized for the preparation of other important carbon materials, such as graphitic carbon nitride by in-situ heating of a suitable molecular precursor. In another case, the Girard’s reagent T (a hydrazide derivative) is treated with fuming nitric acid to afford aqueous-dispersible photoluminescent carbon dots. The choice of Girard’s reagent T as hypergolic fuel is novel in the literature and was merely based on the fact that hydrazine derivatives are common ingredients in hypergolic mixtures. The molecular structure of each combustible fuel (nitrile rubber and Girard’s reagent T) is shown in Scheme 1. Lastly, we show practical applications of the derived functional carbons in waste removal, such as hexavalent chromium and organic dyes.

## 2. Materials and Methods

**Figure 1:**
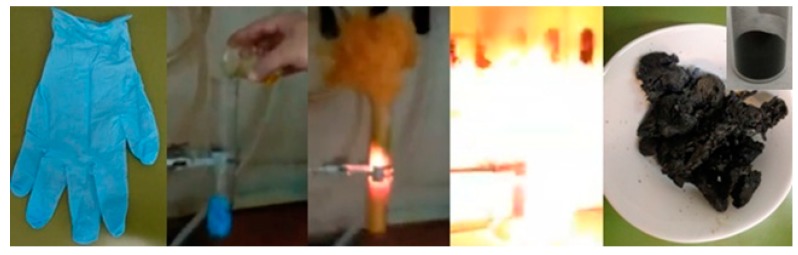
Fuming nitric acid causes ignition of nitrile glove, leading to the formation of carbon foam (far-right photo). Crashing and purification of the foam gives a fine powder composed of carbon nanosheets (far-right inset).

All procedures were performed in a safety hood with a ceramic tile bench. For the synthesis of carbon nanosheets, a medium size disposable nitrile glove (*ca.* 4 g, DURAGLOVE^®^, Penang, Malaysia) was placed inside a large test tube (diameter: 3 cm; length: 20 cm), followed by the quick addition of 10 mL fuming HNO_3_ (100%, Sigma–Aldrich, St. Louis, MO, USA). Upon contact, nitric acid and nitrile glove were spontaneously ignited, releasing brown gases (nitrogen oxides) and an intense flame. After reaction completion, carbon foam was formed (minor residues of unreacted glove were simply cut out). The foam was crashed into a fine powder and copiously washed with dimethylformamide (DMF), tetrahydrofuran (THF), a saturated ethylenediaminetetraacetic acid(EDTA) aqueous solution (EDTA: chelating agent), water, and finally with acetone prior to drying at 80 °C for a day. The obtained powder is thereafter denoted as C-GLOVE (yield: 5%). The overall procedure is visualized in Figure 1.

**Figure 2:**
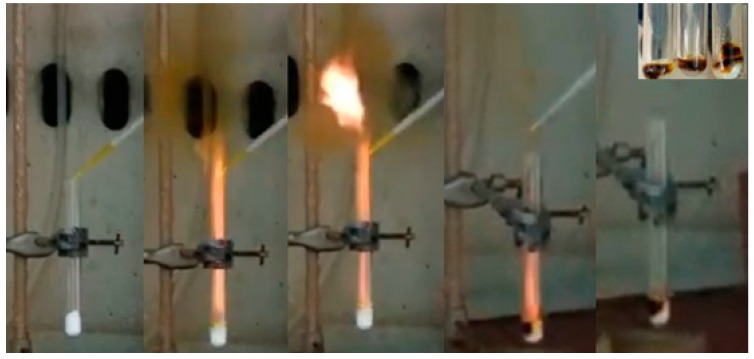
Dropwise addition of fuming nitric acid into Girard’s reagent T ignites the hydrazide derivative, thus resulting in a water-soluble brown residue on the walls of the test tube (see inset).

For the synthesis of carbon dots, 1.5 g Girard’s reagent T (Sigma–Aldrich, St. Louis, MO, USA) was charged in a small test tube (diameter: 1.5 cm; length: 15 cm), followed by the dropwise addition of 1.5 mL fuming nitric acid (100 %, Sigma–Aldrich, St. Louis, MO, USA). The hydrazide derivative was instantly ignited upon contact with the strong oxidizer releasing brown fumes and an intense flame (Figure 2). Simultaneously, a brown residue was formed on the walls of the tube (Figure 2), which was carefully collected and dissolved in a few mL of water. The aqueous solution was filtered off and then placed in a dialysis membrane (molecular weight cut-off MWCO: 14,000) for 48 h—dialysis against water (water was sporadically changed from time to time during dialysis). Finally, the dialyzed solution was filtered off to afford a clear yellowish dispersion of carbon dots in water (yield: 1%).

Powder X-ray diffraction (XRD) was performed using background-free Si wafers and Cu Ka radiation from a Bruker Advance D8 diffractometer (Bruker, Billerica, MA, USA). Raman spectra were recorded with a RM 1000 Renishaw micro-Raman system (Renishaw, Old Town, UK) using a laser excitation line at 532 nm (1 mW). Attenuated total reflection infrared spectroscopy (ATR-IR) measurements were performed using a Jasco IRT-5000 microscope coupled with a FT/IR-4100 spectrometer (Jasco, Easton, MD, USA). The ZnSe prism of the ATR objective had a 250 μm area in contact with the sample. Background was subtracted and the baseline was corrected for all spectra. Ultraviolet-Visible (UV-Vis) spectra were measured in quartz cuvettes with a UV2401 (PC)-Shimadzu spectrophotometer (Shimadzu, Kyoto, Japan). Fluorescence measurements were carried out using a luminescence spectrofluorometer Jasco-8300 (Tokyo, Japan) using a 1 cm path length quartz cuvette. Slit widths with a nominal band pass of 5 nm were used for both excitation and emission ray. The fluorescence emission spectra were recorded from 350 to 650 nm after excitation at different wavelengths, with a scan speed of 100 nm·min^−1^. All emission measurements were taken at room temperature and for every scanned sample, a baseline was recorded and subtracted from the spectrum. X-ray photoelectron spectroscopy (XPS) measurements were performed in an ultra-high vacuum at a base pressure of 4 × 10^−10^ mbar with a SPECS GmbHspectrometer equipped with a monochromatic Mg Kα source (hv = 1253.6 eV) and a Phoibos-100hemispherical analyser (Berlin, Germany). The N_2_ adsorption-desorption isotherms were measured at 77 K on a Sorptomatic 1990, Thermo Finnigan porosimeter (Thermo Finnigan LLC, San Jose, CA, USA). All samples were outgassed at 150 °C for 20 h under vacuum before measurements. Specific surface areas were determined with the Brunauer-Emmett-Teller (BET) method. Atomic force microscopy (AFM) images were collected in tapping mode with a Bruker Multimode 3D Nanoscope (Ted Pella Inc., Redding, CA, USA) using a microfabricated silicon cantilever type TAP-300G, with a tip radius of <10 nm and a force constant of approximately 20–75 N·m^−1^. The transmission electron microscopy (TEM) study of samples deposited on carbon coated copper grids (CF300-CU-UL, carbon square mesh, CU, 300 mesh from Electron Microscopy Science) was performed using the instrument JEM HR-2100, JEOL Ltd., Tokyo, Japan operated at 200 kV in bright-field mode.

## 3. Results and Discussion

### 3.1. Carbon Nanosheets

**Figure 3:**
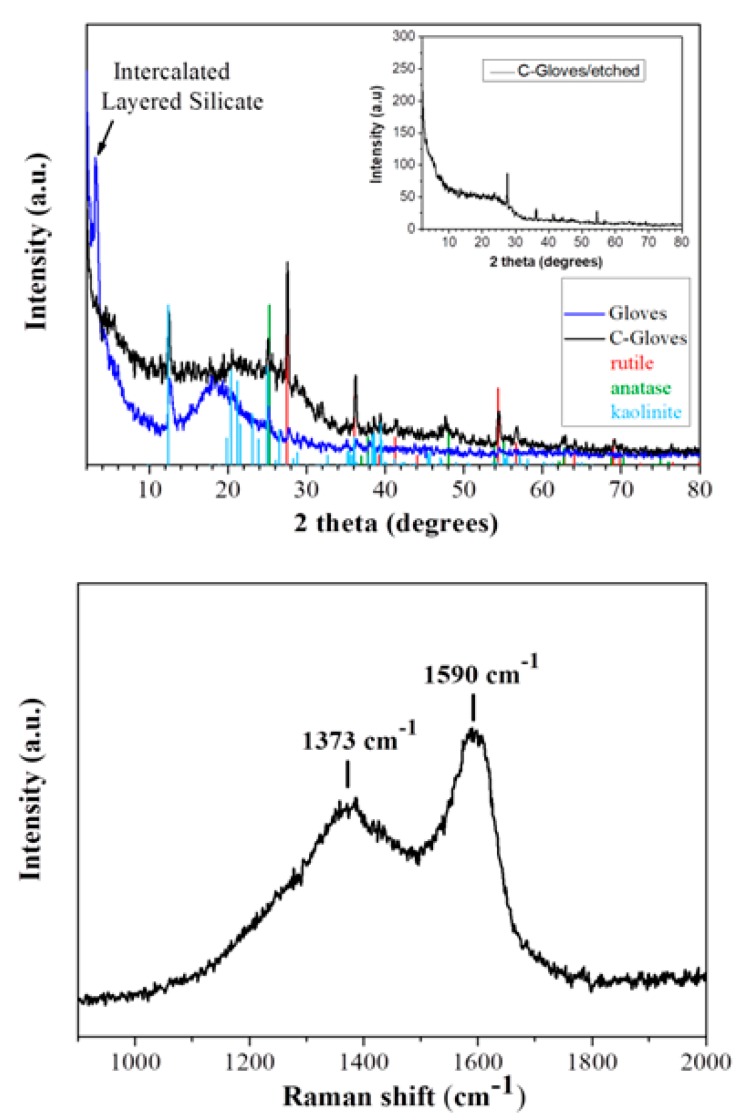
X-ray diffraction (XRD) pattern (**top**) and Raman spectrum (**bottom**) of C-GLOVE. The XRD pattern of the nitrile glove is also presented for comparison (blue line). The XRD pattern of the acid-etched C-GLOVE is given as inset.

The XRD pattern of C-GLOVE exhibited a very broad reflection with *d_002_* value of 3.7–4.1 Å (Figure 3, top). This value is higher than the interlayer distance of crystalline graphite (3.34 Å), thus signalling the formation of amorphous carbon [11]. C-GLOVE also contained several other inorganic phases originating from the nitrile glove. Hence, we could additionally identify the presence of clay (layered silicate) and titania fillers, the latter being added in the glove for mechanical reinforcement and protection against thermal or light degradation [12,13]. Titanium was also qualitatively detected in the glove by X-ray fluorescence (XRF) analysis. Based on thermal gravimetric analysis (TGA) in air, the fillers account for nearly 15% of the C-GLOVE’s composition. It is expected that these residual fillers could further enhance the material’s potentiality in absorption/removal processes or/and photocatalysis. Attempts to remove these fillers by HF (48%) and concentrated HCl (35%) resulted in complete etching of clay but not of titania, especially rutile (Figure 3 top, inset).

**Figure 4:**
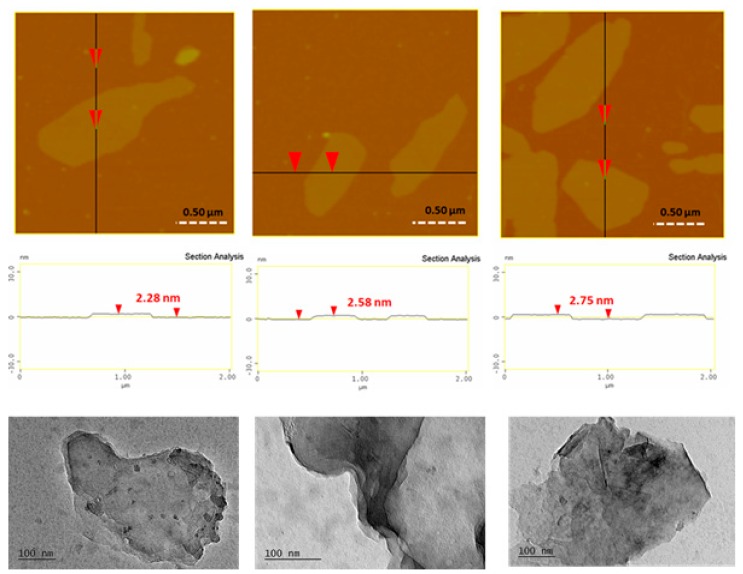
**Top**: Atomic force microscopy (AFM) images of cross-sectional analysis of selected carbon nanosheets in C-GLOVE. **Bottom**: Representative transmission electron microscopy (TEM) images of the sheets.

Similarly with XRD, Raman spectroscopy also pinpointed the formation of amorphous carbon [11], showing broad D (1373 cm^−1^) and G (1590 cm^−1^) bands with a relatively high intensity ratio of *I_D_/I_G_* = 0.7 [14] (Figure 3, bottom). AFM study of the sample revealed the presence of thin nanosheets with an average thickness of 2 to 2.5 nm and micron-sized lateral dimensions (Figure 4, top). The morphology and size of the sheets were confirmed by TEM as well (Figure 4, bottom).

**Figure 5:**
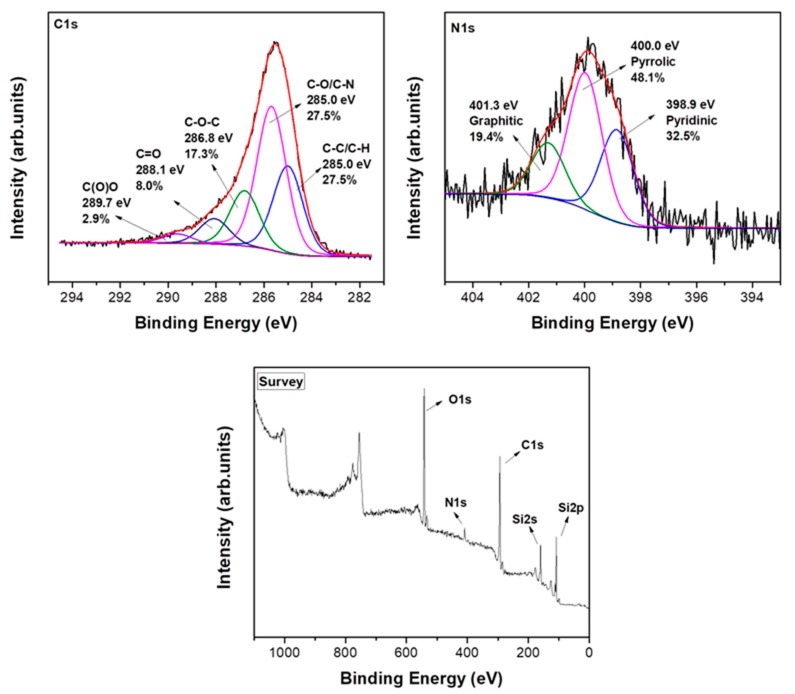
C1s photoelectron spectrum (**left**), N1s photoelectron spectrum (**right**), and X-ray photoelectron spectroscopy (XPS) (**bottom**) survey of C-GLOVE.

The XPS survey spectra of C-GLOVE (Figure 5, bottom) demonstrated the presence of C, O, and N in their composition (the observed silicon peaks are due to the Si substrate used for the measurements). The high resolution C1s XPS spectra revealed the different types of carbon chemical bonds (C–O, C=O, C–O–C, C–N, C–C) present in the samples along with their percentages (Figure 5, left). After fitting on the high resolution N1s XPS spectra, we additionally observed pyridinic, pyrrolic, and graphitic nitrogen species (Figure 5, right) [7]. The C/N ratio was estimated approximately C/N = 15.2.

**Figure 6:**
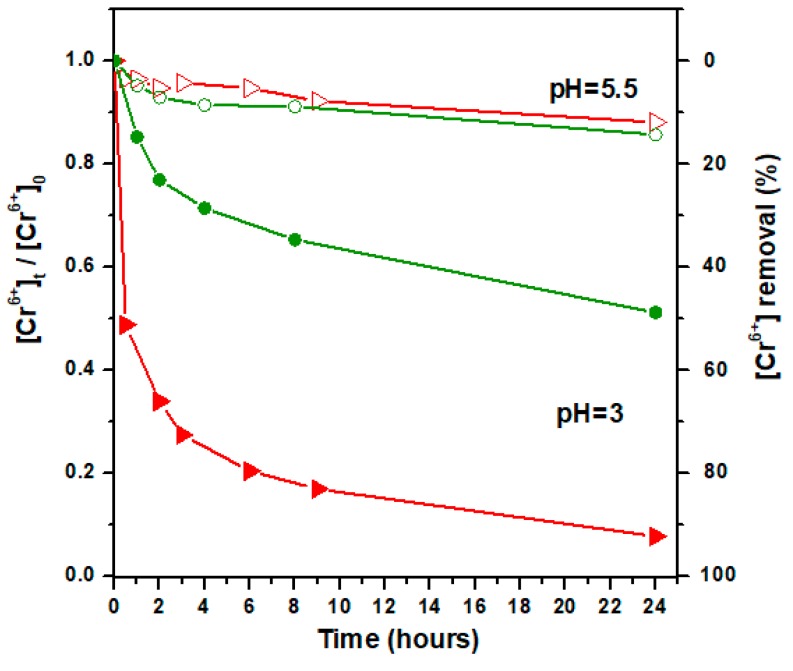
Effect of initial pH value (5.5 and 3) on the Cr (VI) removal efficiency by C-GLOVE (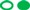) and CMK-3 (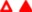).

In one application, the efficiency of C-GLOVE for adsorption of hexavalent chromium ions from aqueous solutions was investigated by conducting sorption kinetic experiments [15]. Figure 6 shows the effectiveness of the material in Cr (VI) removal at initial concentrations equal to 6 mg/L as a function of reaction time for two different pH values (5.5 and 3) at 25 °C. The analogous effectiveness of the well-known ordered mesoporous carbon (CMK-3) measured under same conditions is also shown for comparison [15]. Accordingly, the chromium removal efficiencies of C-GLOVE and CMK-3 after 24 h reaction time at pH 5.5 were determined to be 15% and 12% respectively. On the other hand, at pH 3, the corresponding removal efficiencies reached 50% and 92% for C-GLOVE and CMK-3 respectively. Worth noting, the kinetic data for both carbons were well interpreted by a pseudo-second-order model [15]. Hence, the plots of 1/Cr(VI) versus time (not shown) produced linear plots with correlation coefficients (R^2^) higher than 0.98, indicating that the rate of chromium adsorption by the sorbents fitted well the pseudo second-order model. Based on these plots, the adsorption rate for C-GLOVE was estimated at 1.26 mg∙L^−1^∙h^−1^ versus 2.7 mg∙L^−1^∙h^−1^ for CMK-3 [15]. These results can be rationalized by the much higher specific surface area of CMK-3 compared to C-GLOVE (1650 m^2^/g versus 10 m^2^/g), which in turns favours a greater adsorption.

**Figure 7:**
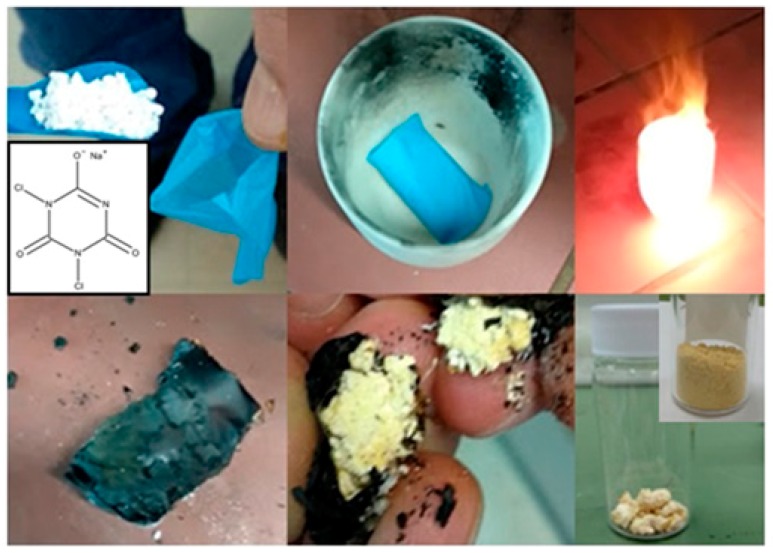
**Top left** to **right bottom**: the middle finger glove tip from a medium size nitrile glove was cut out and stuffed with granular dichloroisocyanuric acid, sodium salt hydrate (top inset structure). The stuffed tip was hermitically folded and placed at the bottom of an alumina crucible. After the quick addition of fuming nitric acid, the nitrile wrap was ignited, providing the necessary heat for the thermal transformation of the enclosed triazine derivative into graphitic carbon nitride. The inner product was mechanically separated from the outer carbon debris in the form of yellow chunks (glass vial at far-right bottom). Purification of the chunks afforded yellow graphitic carbon nitride powder (bottom inset).

In another application, the heat produced from the reaction of nitrile glove with fuming nitric acid was exploited for the rapid thermal transformation of a suitable molecular precursor into graphitic carbon nitride. To this aim, the middle finger glove tip from a medium size nitrile glove was cut out (mass of *ca.* 0.4 g) and stuffed with 1 g of the triazine derivative dichloroisocyanuric acid, sodium salt hydrate (sodium troclosene or pool chlorine, Ezidesaqua, 98%). In general, triazines are considered well-suited precursors towards graphitic carbon nitride by heat [9]. The stuffed tip was hermitically folded and placed at the bottom of an alumina crucible (diameter: 4 cm; height: 5.5 cm). To the crucible, 1 mL of fuming nitric acid was quickly added. The ignition of the nitrile wrap provided the necessary heat for the thermal transformation of the enclosed white-coloured triazine derivative into yellow graphitic carbon nitride, thus acting as a sort of a “heating stove”. After cooling, the inner product was mechanically separated from the outer carbon debris in the form of yellow chunks. The chunks were crashed into a fine powder and washed several times with warm water and acetone prior to drying at room temperature. The overall procedure is visualized in Figure 7.

**Figure 8:**
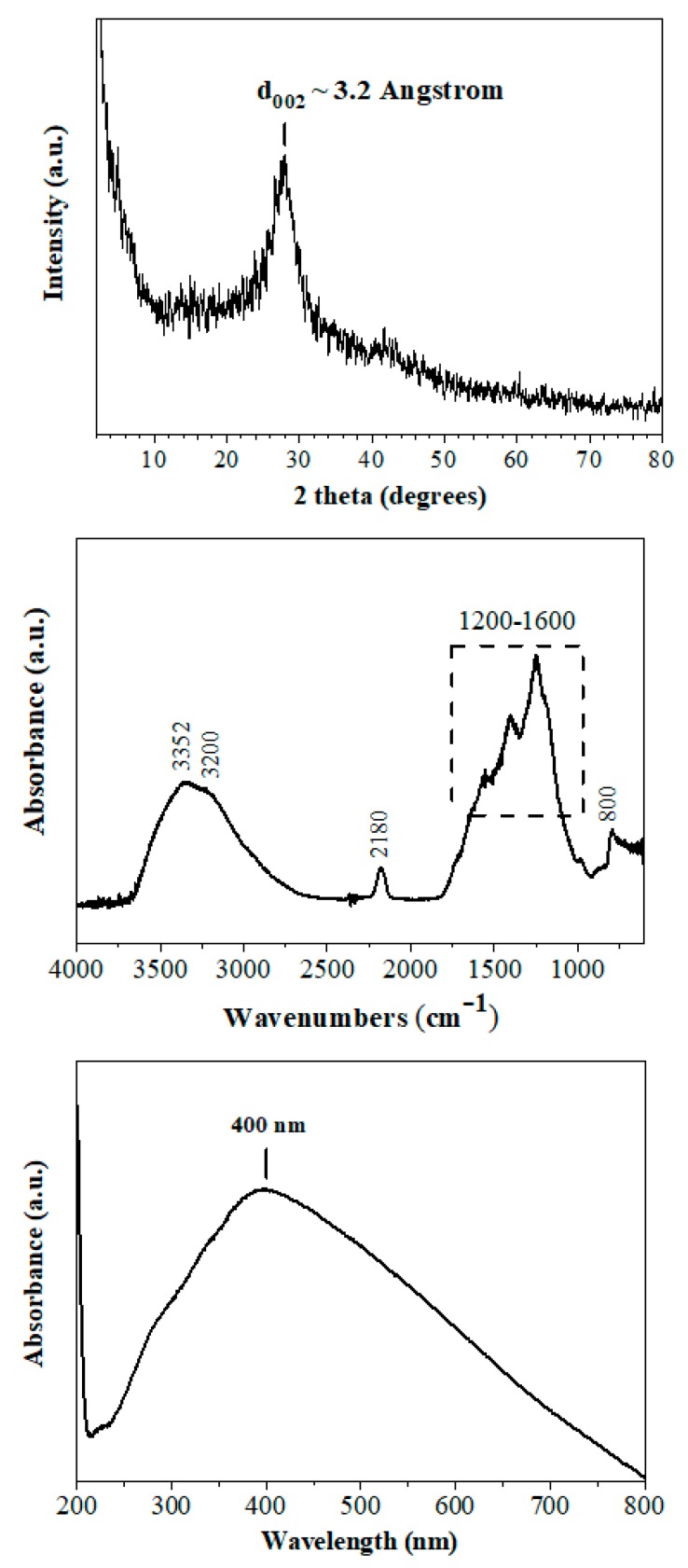
XRD pattern (**top**), Attenuated total reflectance-infrared spectroscopy (ATR-IR) spectrum (**middle**) and ultraviolet-visible (UV-Vis) spectrum (**bottom**); fine solid suspension in ethanol of the as-received graphitic carbon nitride.

**Figure 9:**
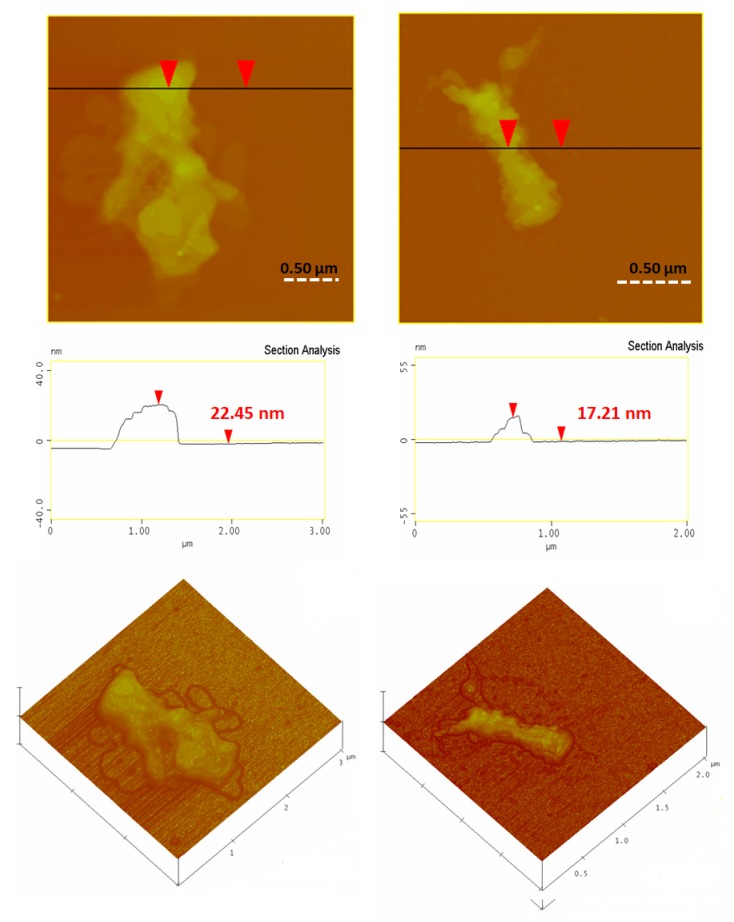
AFM images of cross-sectional analysis and 3D morphologies of graphitic carbon nitride showing the presence of thick multi-layered plates.

The XRD pattern of the yellow powder (Figure 8, top) showed a strong (002) diffraction peak corresponding to an interlayer spacing of *ca.* 3.2 Å, the latter being consistent with graphitic carbon nitride [16,17]. The weak shoulder centered near 2θ = 14° is attributed to the (100) in-plane diffraction peak of the layered solid. The ATR-IR spectrum of the sample (Figure 8, middle) exhibited broad bands from pending –NH_2_ groups at 3352 cm^−1^ and 3200 cm^−1^, while the band at 800 cm^−1^ was assigned to triazine rings [16,17]. The absorption peaks observed in the range 1200–1600 cm^−1^ correspond to the stretching mode of C=N/C-N heterocycles [17]. Lastly, the peak at 2180 cm^−1^ was ascribed to pending –CN triple bonds [18]. The UV-Vis spectrum of the material recorded as a fine solid suspension in ethanol (Figure 8, bottom) displayed a strong absorption centered at 400 nm (violet part of the visible spectrum), thus explaining the yellow colour of the semiconducting solid due to its intrinsic band-gap energy [19]. AFM study revealed the presence of thick plates (*ca.* 20 nm) with multilayered texture near the edges, the latter being typical of layered materials (Figure 9).

**Figure 10:**
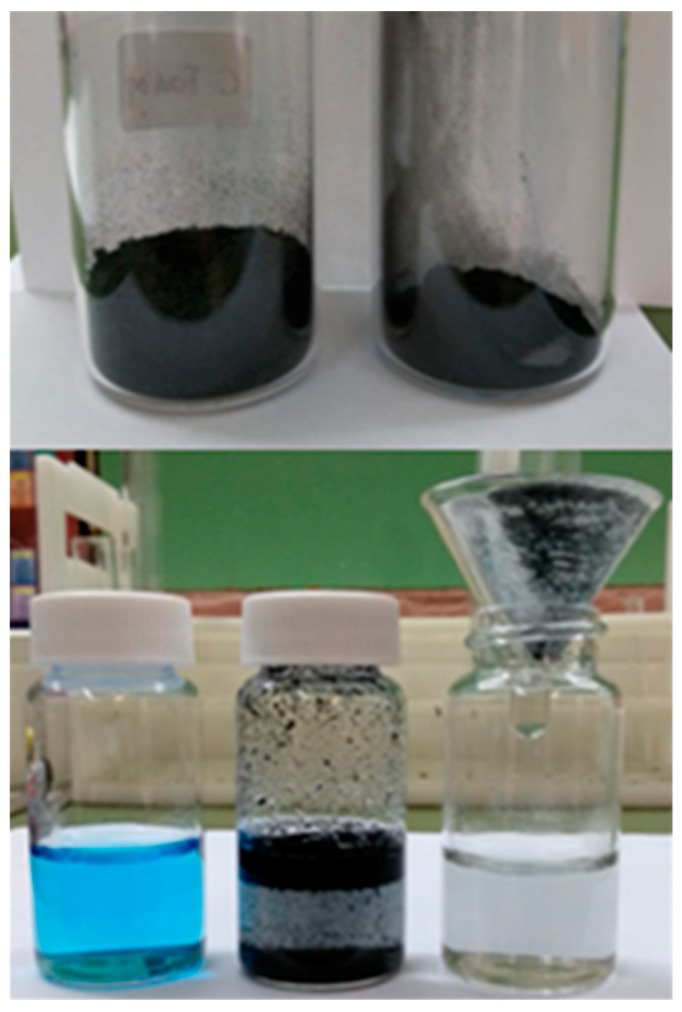
**Top**: The vial on the left shows the volume of the lightweight carbon versus that of commercial charcoal (right vial). Both vials contained 180 mg of the material. **Bottom**: Addition of the lightweight carbon to an aqueous solution of the organic dye methylene blue and subsequent filtration resulted in decolorization of the aqueous phase.

In this same token, another interesting heat source is the aniline-HNO_3_ hypergolic system [4] used here for the carbonization of coffee grains [20,21]. The aniline-HNO_3_ pair instantly ignites towards the formation of complex yet soluble products that, upon washing with water and acetone, leave behind minor carbon residue. Briefly, 0.6 g of instant coffee grains was wetted with 1 mL aniline in a test tube to from a thick paste. At this stage, no reaction between aniline and the coffee grains was observed (e.g., heating or color change). Upon the quick addition of 1 mL of fuming nitric acid, the mixture ignited with short delay to produce fluffy carbon on the walls and the rim of the test tube. Apparently, the energy released from the spontaneous ignition of the hypergolic mixture provided the necessary heat for the carbonization of the coffee grains. The product was scratched off, washed thoroughly with water and acetone, and finally dried at 80 °C. An amorphous, lightweight carbon powder was obtained, which was able to decolorize dye-contaminated water (Figure 10).

**Figure 11:**
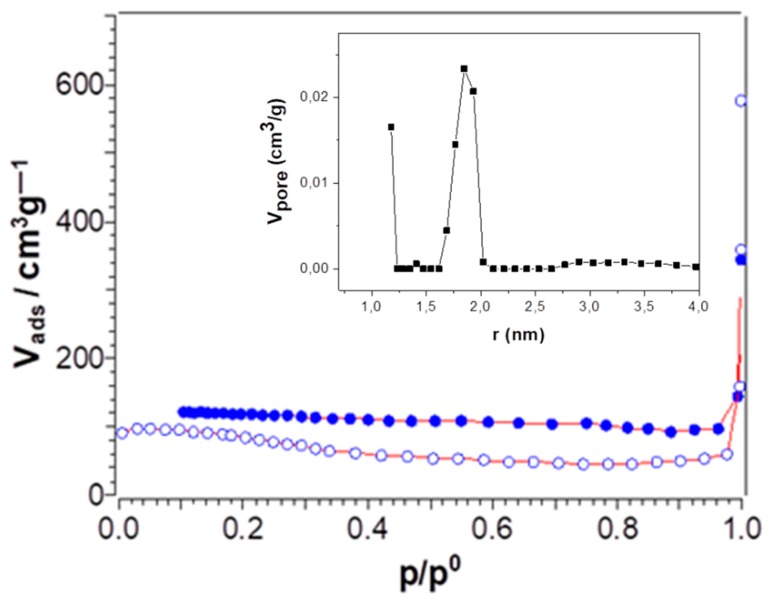
N_2_ adsorption-desorption isotherms and pore size distribution (inset) for the coffee-derived carbon.

The N_2_ adsorption-desorption isotherms of the coffee-derived carbon are shown in Figure 11. The sample exhibited a mixed I and II type corresponding to a microporous structure with an indefinite multi-layer formation after completion of the monolayer. The BET method was applied for calculation of specific surface area; the material’s S_BET_ was calculated as ~288 m^2^·g^−1^. From the pore size distribution obtained using DFT simulation, the sample possessed mainly micropores with average size, ~1.9 nm. Finally, the micropore volume was found to be ~0.12 cm^3^·g^−1^ and the cumulative pore volume ~0.19 cm^3^·g^−1^.

### 3.2. Photoluminescent Carbon Dots

**Figure 12:**
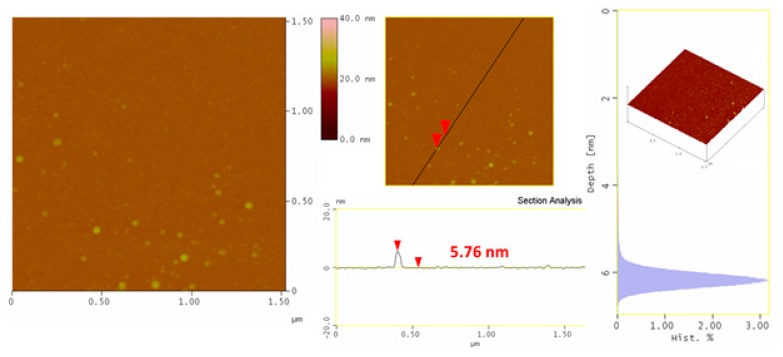
Topography images of carbon dots (section-analysis, 3D morphology, and depth-analysis histograms are included).

The ignition of nitrile glove by fuming nitric acid is considered a classic demonstration experiment in the area of hypergolic reactions that, as shown here, resulted in carbon nanosheets. In an effort to demonstrate the generic character of the concept towards the synthesis of other types of functional carbon materials, we also present here the formation of photoluminescent carbon dots by the ignition of Girard’s reagent T with fuming nitric acid. To the best of our knowledge, the Girard’s reagent T-HNO_3_ hypergolic system is reported for the first time in the literature. The reaction led to a hydrophilic brown residue that after dialysis against water gave an aqueous dispersion of carbon dots with a spherical morphology and mean size of 5 nm based on AFM (Figure 12). The hydrophilic nature of the dots is ascribed to the quaternary ammonium type of structure of the starting hydrazide derivative [22].

**Figure 13:**
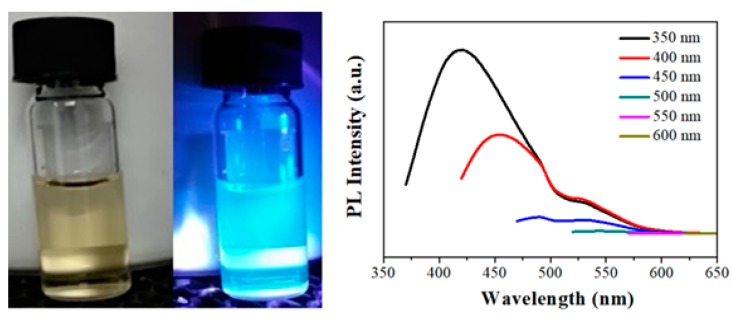
**Left**: aqueous dispersion of carbon dots under natural and UV light. **Right**: fluorescence emission spectra of the dots at different excitation wavelengths (*λ_ex_* as inset).

Interestingly, the dispersed dots fluoresced in the visible region, as shown in Figure 13 (left). With respect to the fluorescence spectra (Figure 13, right), when the excitation wavelength varied from 350 to 600 nm, the spectra red shifted and the fluorescence intensity decreased gradually. Such excitation-depended emission behaviour is consistent with photoluminescent carbon dots [10,22,23]. The fluorescence quantum yield was roughly estimated to be 1–2%.

## 4. Conclusions

This work shows a new synthesis method for functional carbon materials (carbon nanosheets, graphitic carbon nitride, and photoluminescent carbon dots) based on hypergolic mixtures consisting of a combustible organic solid and fuming nitric acid as strong oxidizer. In all instances, the carbon phase formation was fast, spontaneous, and exothermic at ambient conditions, making hypergolic mixtures an interesting new source of carbon nanomaterial. Some practical uses of the derived functional carbons included here are hexavalent chromium removal and decolorization of water-soluble organic dyes. These findings build upon previous results from our group on carbon materials obtained at ambient conditions from pyrophoric lithium dialkylamides salts (e.g., carbon nanosheets) or hypergolic acetylene-chlorine mixtures (e.g., synthetic graphite) [6,7]. Some additional paradigms for future study include hypergolic mixtures containing a combustible organic compound and sodium peroxide (source of highly concentrated H_2_O_2_), and manganese heptoxide or chromyl chloride as strong oxidizers (see *Bretherick’s Handbook of Reactive Chemical Hazards*).
